# Genetic Organisation, Mobility and Predicted Functions of Genes on Integrated, Mobile Genetic Elements in Sequenced Strains of *Clostridium difficile*

**DOI:** 10.1371/journal.pone.0023014

**Published:** 2011-08-18

**Authors:** Michael S. M. Brouwer, Philip J. Warburton, Adam P. Roberts, Peter Mullany, Elaine Allan

**Affiliations:** Department of Microbial Diseases, UCL Eastman Dental Institute, University College London, London, United Kingdom; Baylor College of Medicine, United States of America

## Abstract

**Background:**

*Clostridium difficile* is the leading cause of hospital-associated diarrhoea in the US and Europe. Recently the incidence of *C. difficile*-associated disease has risen dramatically and concomitantly with the emergence of ‘hypervirulent’ strains associated with more severe disease and increased mortality. *C. difficile* contains numerous mobile genetic elements, resulting in the potential for a highly plastic genome. In the first sequenced strain, 630, there is one proven conjugative transposon (CTn), Tn*5397*, and six putative CTns (CTn*1*, CTn*2* and CTn*4*-*7*), of which, CTn*4* and CTn*5* were capable of excision. In the second sequenced strain, R20291, two further CTns were described.

**Results:**

CTn*1*, CTn*2* CTn*4*, CTn*5* and CTn*7* were shown to excise from the genome of strain 630 and transfer to strain CD37. A putative CTn from R20291, misleadingly termed a phage island previously, was shown to excise and to contain three putative mobilisable transposons, one of which was capable of excision. *In silico* probing of *C. difficile* genome sequences with recombinase gene fragments identified new putative conjugative and mobilisable transposons related to the elements in strains 630 and R20291. CTn*5*-like elements were described occupying different insertion sites in different strains, CTn*1*-like elements that have lost the ability to excise in some ribotype 027 strains were described and one strain was shown to contain CTn*5*-like and CTn*7*-like elements arranged in tandem. Additionally, using bioinformatics, we updated previous gene annotations and predicted novel functions for the accessory gene products on these new elements.

**Conclusions:**

The genomes of the *C. difficile* strains examined contain highly related CTns suggesting recent horizontal gene transfer. Several elements were capable of excision and conjugative transfer. The presence of antibiotic resistance genes and genes predicted to promote adaptation to the intestinal environment suggests that CTns play a role in the interaction of *C. difficile* with its human host.

## Introduction

*Clostridium difficile* is an anaerobic spore-forming bacterium that can be part of the normal gut flora in healthy individuals [1]. Antibiotic treatment disrupts the microbial community in the gut, providing an opportunity for *C. difficile* to compete with the other species and induce disease by toxin production. The *C. difficile* toxins affect gut epithelial cells and result in symptoms ranging from mild diarrhoea to the potentially fatal condition, pseudo-membranous colitis [2]. Although toxins A and B are the main virulence factors known for *C. difficile* [3], the role of other factors, such as adhesins and other toxins, and the mechanisms by which these virulence factors are regulated, remain to be determined.

Once considered relatively rare, there has been a global increase in the incidence of *C. difficile*-associated disease (CDAD) since the turn of the century. A number of explanations for the increase have been proposed including the emergence of so-called ‘hypervirulent’ strains, especially those belonging to ribotype 027/North American PFGE type, NAP1 [4] which are associated with more severe disease, higher rates of mortality, higher relapse rates and increased resistance to fluoroquinolones [5]. Whilst ribotype 027/NAP1 strains have received much attention, in other countries different ribotypes have emerged (eg., 078) and these may also have the potential to cause severe disease [6], [7].

*C. difficile* 630, a strain isolated in 1982 from a hospital patient with severe pseudomembraneous colitis was the first strain to be fully sequenced [8]. It had previously been shown to contain the conjugative transposon Tn*5397* [9], also referred to as CTn*3* [8], and the mobile element Tn*5398* [10], providing the host with tetracycline- and erythromycin-resistance, respectively. Full genome annotation revealed that strain 630 contains additional mobile genetic elements including bacteriophages, IS elements, IStrons (a chimera of an IS element and a group I intron) and putative conjugative transposons: CTn*1*, CTn*2*, CTn*4*, CTn*5*, CTn*6* and CTn*7* [8]. These elements have recently been given the prefix CDCTn, however no justification for this renaming was presented [11]. *C. difficile* strain R20291 (ribotype 027), the index isolate in an outbreak at Stoke Mandeville Hospital, UK in 2006, has recently been sequenced and was also shown to contain a large number of putative mobile genetic elements, of which two putative conjugative transposons differ significantly from related elements in 630 [12].

Conjugative transposons are mobile genetic elements capable of integration and excision from the host genome and conjugational transfer by means of proteins encoded by genes on the element [13]. Additionally, they contain accessory genes that are not involved in transfer and which often encode functions that contribute to the environmental adaptability of the host cell, commonly antibiotic resistance-conferring proteins [14]. In this work, searches of the *C. difficile* genome sequences available at NCBI using a library of recombination genes identified new putative conjugative and mobilisable transposons. We have examined the genome sequences of ten *C. difficile* strains including R20291, a recent UK ribotype 027/NAP1 isolate, four recent ribotype 027 isolates, one recent 078 ribotype isolate and one ribotype 001 isolate all from Quebec, Canada, as well as two historical ribotype 027 strains isolated in France and Canada (CD196 and QCD-76W55, respectively), The ability of the newly discovered elements to excise from the host genome was investigated, as was the mobility of the previously described elements in strains 630 and R20291. In addition, using a selection of bioinformatics programs, we predict the potential function of some of the accessory gene products carried by these transposons.

## Results

### Excision of the putative conjugative transposons in strain 630

**Figure 1:**
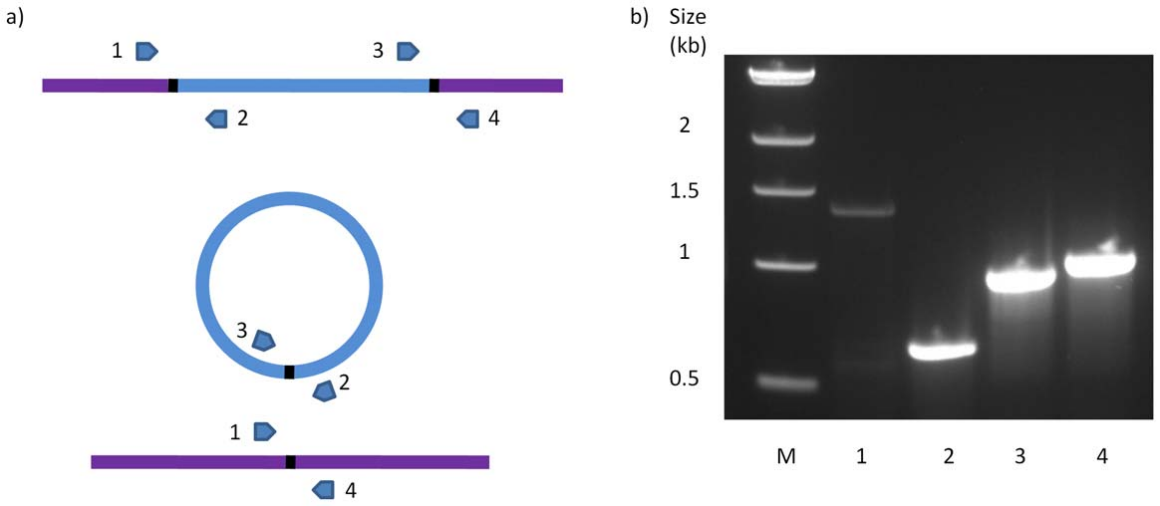
Detection of excision of the elements from the genome. a) Schematic of primer binding sites on the element and the genome. The chromosomal region is shown in lilac and the element in blue, the circular form of the element is also shown (centre), as is the regenerated target after excision (bottom). Oligonucleotide primers and their direction of priming are represented by arrows. Primer pair 1+4 will detect the empty target site, primer pair 2+3 will detect the circular form of the element, primer pairs 1+2 and 3+4 for detection of the junctions between the genome and the element. b) PCR products run on 1% agarose gel for CTn*5* excision from 630 genomic DNA. Lane 1; primers 2+3, lane 2; primers 1+4, lane 3; primers 3+4, lane 4; primers 1+2.

**Figure 2:**
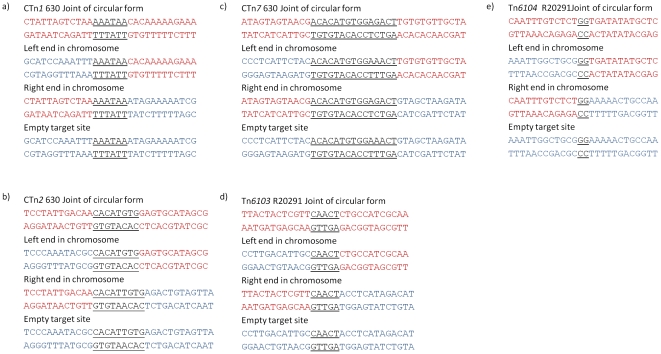
Sequences of the joints of circular intermediates element-genome junctions and regenerated target sites after excision. Sequence in red is part of the transposons, blue is part of the chromosome. Terminal repeats are underlined. a) CTn*1*, b) CTn*2*, c) CTn*7*, d) Tn*6103* in R20291, e) Tn*6104* in R20291 sequenced across the circular joint and left and right ends in the target site only.

**Figure 3:**
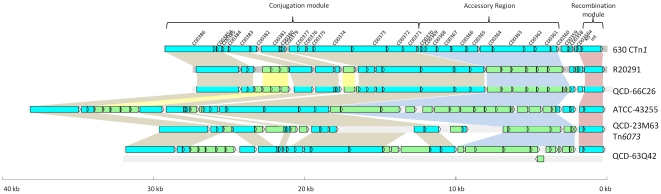
Schematic representation of CTn*1*-like elements in *C. difficile* strains. Blue ORFs have homologues present in CTn*1* in strain 630, green ORFs are not present in CTn*1*. Coloured boxes show regions of homology: red boxes show recombination modules, blue boxes show accessory genes, brown boxes show conjugation modules, yellow boxes show homologues that are not present in 630 CTn*1*. The element in strain QCD-66C26 is representative of the elements in strains QCD-32G58, QCD-37X79, QCD-76W55 and QCD-97B34.

Strain 630 has 6 putative conjugative transposons: CTn*1*, CTn*2*, CTn*4*, CTn*5*, CTn*6* and CTn*7*, of which, CTn*4* and CTn*5* have been shown to excise from the genome [8]. In order to determine whether the other putative elements are capable of excision, specific oligonucleotide pairs were used to PCR amplify the element-genome junctions, the joints of the element in a circular form (the transposition and conjugal intermediate) and the regenerated target site after excision. Figure 1 shows the experimental details. PCR products were produced with CTn*1*, CTn*2* and CTn*7*, and sequencing showed the element-genome junction, the empty target site in the chromosome after excision and the joint sequence in the circular molecules (Figure 2). This analysis allowed the ends of the various elements to be defined (Figure 2 and Table 1). CTn*1* was delineated by a 6-bp direct repeat, one copy of which was present in the joint of the circular form and in the empty target after excision of the element (Figure 2a). CTn*1* contains a tyrosine integrase (CD0355) which, together with the excisionase (CD0356) (Figure 3), is likely to be responsible for excision.

**Table 1:** Properties of putative conjugative and mobilizable transposons in *C. difficile*.

Strain	Element	Genome excision	Properties	CDS start	CDS stop	Size (kb)	Percent G+C	Transfer frequency (σ)	Genbank accession number (if appropriate)
**630**	CTn*1*	Yes		CD0355	CD0386	28.9	38.6	7.0*10^−9^ (9.0*10^−9^), 2.0*10^−8^ (1.7*10^−8^)	
	CTn*2*	Yes		CD0408	CD0436	42.2	35.1	2.1*10^−4^ (5.5*10^−4^)	
	Tn*5397*	Yes	Tc^R^	CD0496	CD0511	20.7	38.3		
	CTn*4*	Yes		CD1091	CD1118	30.5	46.6	2.3*10^−6^ (4.2*10^−6^)	
	CTn*5*	Yes		CD1845	CD1878a	45.6	32.7	2.8*10^−5^ (2.6*10^−5^)	
	CTn*6*	No		CD3326	CD3348	21.3	42.8		
	CTn*7*	Yes		CD3370	CD3392	29.2	40.9	9.6*10^−9^ (4.4*10^−9^)	
	Tn*5398*	ND	Erm^R^	CD2001	CD2010b	9.6	35.4		
**R20291**	CTn*1*-like	No		3453	3475	27.2	39.9		
	Tn*6103*	Yes		1740	1809	84.9	41.2	<10^−11^	BK008007, JF422666
	Tn*6104*	Yes		1744	1764	15.6	48.2		
	Tn*6105*	No		1765	1775	15.8	49.8		
	Tn*6106*	No		1777	1788	11.3	49.9		
**CD196**	CTn*1*-like	ND		3407	3429	27.2	39.7		
**QCD-23M63**	Tn*6073*	Yes		3137	2962	29.1	39.3		BK008006, JF422665
	CTn*4*-like	ND		4815	4970	25.0	45.0		
	Tn*6107*	Yes		16791	16991	50.5	33.5		BK008008, JF422667
**QCD-63Q42**	CTn*1*-like	NA		7272	7692	71.7	42.0		
	CTn*1*-like	NA		9214	9039	30.9	39.7		
	CTn*5*-like	NA		17051	17266	52.2	32.4		
	Tn*6115*	NA		7732	7787	13.6	47.2		
	CTn*7*-like	NA		17276	17431	29.6	40.5		
**ATCC-43255**	CTn*1*-like	No		6310	6090	38.5	37.7		
	Tn*5398*-like	ND		10502	10527	5.4	36.9		
**QCD-37X79**	CTn*1*-like	NA		18585	18425	27.2	39.4		
	CTn*5*-like	NA		16948	17243	58.0	37.8		
**QCD-66C26**	CTn*1*-like	No		18275	18120	27.2	39.4		
	Tn*6110*	Yes		16663	16958	58.0	37.8		BK008009, JF422668
**QCD-32G58**	CTn*1*-like	No		4196	4166	27.2	39.4		
	Tn*6111*	Yes		2165	3914	53.4†	37.9†		JF422669
**QCD-76W55**	CTn*1*-like	NA		18479	18319	27.2	39.6		
**QCD-97B34**	CTn*1*-like	NA		18008	17853	27.2	39.5		

Tc: tetracycline, Erm: erythromycin, ^S^: sensitive, ^R^: resistant. Genome excision ND = not determined, NA = not available. σ: standard deviation.

† = Sequence of the element was not joined in assembled genome, size and G+C percentage are estimates. The sequences of novel circular intermediates were deposited in Genbank with the accession numbers given. Transfer frequency is calculated as the number of transconjugant cells per donor cell. Two transfer frequencies are reported for CTn*1* marked with the Clostron in two different genes, CD0364 and CD0386, respectively.

**Figure 4:**
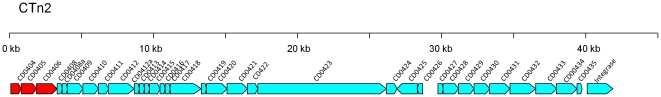
Schematic representation of CTn*2* in strain 630. ORFs in blue are now predicted to be part of CTn*2*, ORFs shown in red were previously thought to be part of the element [8] but were present in the target site after excision (see text for more details).

The boundary of CTn*2* is defined by an imperfect direct repeat with the 8-bp sequence on the left end of the element present in the joint of the circular form, and the 9-bp sequence on the right end of the element left in the empty target site after excision (Figure 2b). There is one large serine recombinase (CD0436) in this element which is likely to be responsible for excision, although the exact mechanism requires further investigation. Our analysis also shows that CD0404 to CD0406 are not part of the transposon and remain in the chromosome after excision (Figure 4), demonstrating that the ends of CTn*2* are not as previously reported [8].

CTn*7* is flanked by 15-bp imperfect direct repeats (Figure 2c). The sequence on the right end of the element is present in the joint of the circular form and the sequence on the left end of the element is found in the empty target site after excision. The element contains one large serine recombinase (CD3370) which is likely to be responsible for the excision reaction.

CTn*6* is the only putative conjugative transposon in strain 630 for which no joint of a circular form or empty target site could be detected. Results are summarised in Table 1.

### Genetic organisation of conjugative transposons in strain R20291

Strain R20291 (ribotype 027) contains two putative conjugative transposons that are variants of elements found in strain 630 [12]. One of these has a structure comparable to CTn*1* in strain 630, the main difference being the accessory module of the elements (Figure 3). Excluding the accessory module, the remainder of the two elements show at least 82% nucleotide sequence identity. We could not detect excision of the element in R20291, possibly because of a deletion of three ORFs, including the putative *xis* gene, compared to CTn*1* in 630 (Figure 3). Furthermore, this element was found to have integrated into a different site within the genome of R20291 when compared to 630, integrating between ORFs 3452 and 3476 in R20291, genes encoding a hypothetical protein and a putative transcriptional regulator, respectively (ORFs 3452 and 3476 in R20291 are homologues of CD3614 and CD3615 in strain 630). In 630, CTn*1* is integrated into CD0354, a gene encoding a hypothetical protein; an uninterrupted homologue of this gene is present in R20291.

**Figure 5:**
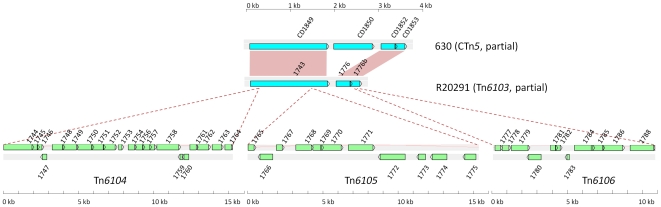
Schematic representation of Tn*6104*, Tn*6105* and Tn*6106* in Tn*6103*. Comparison of CD1849–CD1853 of CTn*5* in strain 630 and the homologous region of Tn*6103* in R20291 (see Figure 8 for a diagram of the whole of CTn5). Homologous genes are shown by red boxes. The three separate insertions in Tn*6103*, are shown by dotted lines.

The second putative conjugative transposon in R20291, has been previously reported as CTn*027* and an insertion in the element was erroneously called the Stoke Mandeville phage island, SMPI [12]. There is no evidence of a phage within this element or that the element itself is a prophage, and we renamed it Tn*6103* as it fits the criteria for a conjugative transposon according to the transposon registry guidelines [15]. This element is similar to CTn*5* in strain 630, having at least 85% nucleotide identity along most of its length; however, it contains three insertions which are probably mobilizable transposons (see below), two of which are inserted within ORF 1743 and one within ORF 1776. These elements have been named Tn*6104*, Tn*6105*, and Tn*6106* (Figure 5). All three elements contain a recombinase gene, however, excision and circularisation has been demonstrated only for Tn*6104* as well as the composite element itself, Tn*6103* (Figure 2e). Tn*6104* contains 21 orfs and is flanked by a 2-bp direct repeat which is also present in the circular form of the element and the empty target site after excision (Figure 2e). The recombinase of this element, ORF 1744, is a member of the family of large serine recombinases and is related to TnpX from the mobilisable transposons Tn*4451* and Tn*4453* of *Clostridium perfringens* and *C. difficile*, respectively [16], [17]. TnpX also has a 2-bp target site [18]. Tn*6104* has another gene product (encoded by ORF 1745) which is 48% identical at the amino acid level to TnpV of Tn*4451*, postulated to be involved in excision of Tn*4451* based on homology with λ Xis [18]. Another similarity between Tn*6104* and Tn*4451* is the *mobA/mobL* mobilisation gene (ORF 1758) which is present in the same orientation at the right end of the element. In contrast to the single accessory gene, *catP* in Tn*4451*, Tn*6104* contains several accessory genes with the potential to encode a putative transcriptional regulator (ORF 1747), a two component regulatory system (ORFs 1748 and 1749), an ABC transporter (ORFs 1750, 1751 and 1752), three sigma factor-like proteins (ORFs 1754, 1755 and 1756), a putative toxin-antitoxin system (ORFs 1759 and 1760) and a phage-associated protein (ORF 1762) (see Table S1 and section on predicted accessory gene function below).

Excision of Tn*6105* and Tn*6106* was not detected. Tn*6105* consists of 11 ORFs and contains two putative large serine recombinase genes situated in the centre of the element (ORFs 1771 & 1772, Figure 5). Other putative genes are *tnpV* (ORF 1765), as well as a mobilisation protein (ORF 1768), a predicted sigma factor (ORF 1773) and a predicted orphan response regulator (ORF 1775) (Table S1). Tn*6106* consists of 11 ORFs and contains a single large serine recombinase gene on the right side of the element (ORF 1788, Figure 5). Other genes encode a TnpV homologue (ORF 1777), a predicted mobilisation protein (ORF 1784) and a predicted transcriptional regulator (ORF 1778) (Table S1).

Tn*6103* itself is flanked by perfect 5-bp direct repeats and one of these is present in the joint of the circular form and in the empty target site in the genome after excision, identical to CTn*5* in 630 (Figure 2d) [8]. The element contains a large serine recombinase which is likely to be responsible for its excision.

### The majority of the CTns are capable of conjugative transfer

To study the transfer of the putative conjugative transposons, the ClosTron system [19] was retargeted to ORFs within the accessory module of each element that were predicted not to be involved in conjugation.

The transposons marked with the ClosTron could still excise from the genome, as determined by PCR. Filter mating assays were performed using strains containing a marked element as donor and *C. difficile* CD37 as recipient. Transconjugants were screened by PCR for the presence of the inserted ClosTron, as well as the absence of the PaLoc (to confirm them as strain CD37). All six marked elements from strain 630 transferred into the recipient strain CD37 at frequencies between 10^−4^ and 10^−9^ (Table 1). Transfer of Tn*6103* from strain R20291 to CD37 was not detected indicating either that the element cannot transfer into CD37, or does so at a transfer frequency below the detection limit.

### Identification of putative conjugative and mobilisable transposons in other sequenced *C. difficile* genomes

The nucleotide sequences of the genes encoding serine- or tyrosine-based recombinases associated with conjugative transposons, plus those phylogenetically-related genes present in bacterial genomes, were downloaded from Genbank at the NCBI (see materials and methods for more details). These sequences, together with the sequences of the ORFs present on the CTns in strain 630, were used in BLAST searches of the *C. difficile* genome sequences available at NCBI. This analysis allowed the identification of the novel putative mobile elements summarised in Table 1. The Artemis Comparison Tool (ACT) [20] was used to compare the novel elements to the previously identified conjugative transposons in strains 630 and R20291. ORFs annotated as hypothetical proteins in genome sequencing projects were analysed using selected bioinformatics programs (see materials and methods) (full data provided in Table S1).

### CTn*1*-like elements

Diverse variants of CTn*1* were found in all the *C. difficile* genomes that were searched (Table 1). All strains contain an element sharing 98% nucleotide identity with the element described in R20291 (see above) (Figure 3). Additionally, all of these elements are present in the same target site as the CTn*1*-like element in R20291 and, in common with this element, do not have an excisionase (*xis*) homologue. The elements of strains QCD-32G58 and QCD-66C26 were analysed for excision from the genome but no PCR product for the joint of the target site or the circular form could be amplified, presumably due to the lack of a functional Xis. All these strains are ribotype 027/NAP1 suggesting that a CTn*1*-like element transferred into the ancestor of the modern ribotype 027/NAP1 strains where it suffered a deletion of *xis*, fixing it within the host chromosome. The accessory modules of these elements contain predicted ABC transporters. Other accessory gene products encoded by these elements are shown in Table S1 and some are discussed in the section on accessory gene function below.

**Figure 6:**
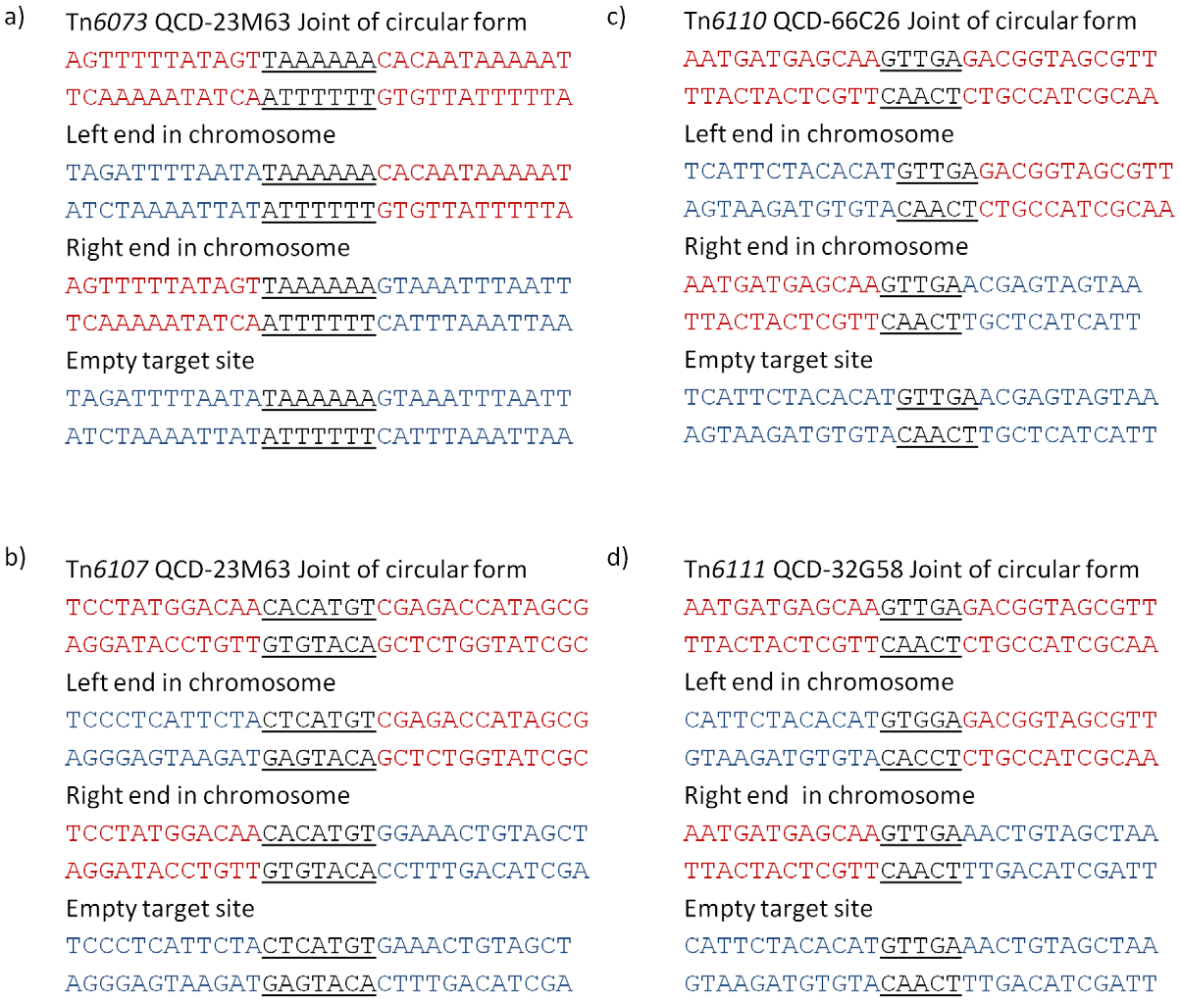
Sequences of the joints of circular intermediates element-genome junctions and regenerated target sites after excision. Sequence in red is part of the transposons, blue is part of the chromosome. Terminal repeats are underlined. a) Tn*6073* QCD-23M63, b) Tn*6107* QCD-23M63, c) Tn*6110* QCD-66C26, d) Tn*6111* QCD-32G58.

Strain QCD-23M63 (ribotype 078) contains a CTn*1*-like element (75–99% nucleotide sequence identity with CTn*1* in 630, excluding the accessory module) which we have named Tn*6073* (Figure 3). The element is flanked by 7-bp direct repeats, one of which is present in the joint of the circular form and one in the empty target site in the genome after excision (Figure 6a). The element contains a tyrosine recombinase and excisionase which together are likely to be responsible for its excision. Tn*6073* is located in a different target site from CTn*1* in 630: it is inserted between homologues of the 630 genes CD0651 and CD0652, which are predicted to encode a membrane protein and a transcriptional regulator, respectively. The accessory module of the element consists of genes encoding a predicted N-terminal hydrolase, a sigma factor and an ABC transporter (see Table S1 and section on accessory gene function below). Compared to CTn*1* in 630, there are three insertions in the conjugation module which contain hypothetical genes (Figure 3).

**Figure 7:**
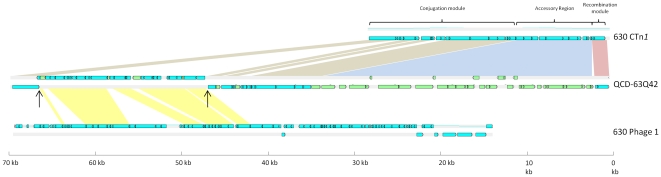
Schematic representation of the CTn*1*-like element in *C. difficile* QCD-63Q42. Blue ORFs have homologues present in CTn*1* in strain 630, green ORFs are not present in CTn*1*. Coloured boxes show regions of homology: red boxes show recombination modules, blue boxes show accessory genes, brown boxes show conjugation modules, yellow boxes show homologues that are not present in CTn*1* in strain 630. Comparison of CTn*1* in 630, CTn*1*-like element in QCD-63Q42 and phage 1 of 630. Black arrows indicate gaps in the DNA sequence.

Strain QCD-63Q42 (ribotype 001/NAP2) contains two CTn*1*-like elements, the first of which is inserted between homologues of CD1565 and CD1564 and has a minimum of 73% nucleotide sequence identity (excluding insertions) with CTn*1* of strain 630. An insertion of approximately 20-kb between homologues of CD0386 and CD0383 in this CTn*1*-like element contains a sequence with on average 92% sequence identity with prophage 1 of strain 630 [8]. However, until the sequence gaps either side of the partial phage are filled, it is not possible to say unequivocally that this is the actual insertion site of this element (Figure 7). Another interesting feature of this element is the fact that the accessory module is 33-kb (compared to 9.5-kb in strain 630) and includes genes predicted by bioinformatics analysis to encode an ABC transporter, two sigma factors and a transcriptional regulator (Table S1). The second CTn*1*-like element in strain QCD-63Q42 is inserted between homologues of CD1807 and CD1806. Two insertions in the conjugation module include genes encoding a putative alpha/beta hydrolase, a lactoylglutathione lyase, a group II intron reverse transcriptase, as well as several hypothetical proteins. The accessory module contains two putative ABC-transporter genes and four transcriptional regulators of which one is predicted to be a sigma factor, as well as several hypothetical proteins (Table S1). Both elements contain an intact *xis* homologue and a complete tyrosine recombinase suggesting that they can excise from the genome, although this has not been investigated.

Strain ATCC-43255 (formerly VPI 10463) contains a CTn*1*-like element (at least 73% nucleotide sequence identity with CTn*1* from 630 excluding the accessory module) inserted between homologues of the 630 genes, CD1234 and CD1235, two hypothetical genes within the prophage-like *skin* ^CD^ element which is itself inserted into the sigma K (σ^k^) gene, involved in sporulation [21]. The *skin* ^CD^ element was shown to excise from σ^k^ during sporulation, forming a circular molecule. However the *skin* ^CD^ element itself was not characterised in that study and therefore the presence of the CTn*1*-like element within *skin* ^CD^ was not detected. We could not detect excision of the CTn*1*-like element from *skin* ^CD^ by PCR. Taken together, our results and those of Haraldsen *et al* [21] demonstrate that the presence of the CTn*1*-like element within *skin* ^CD^ does not prevent its excision and does not prevent sporulation in this strain.

### CTn*5*-like elements

**Figure 8:**
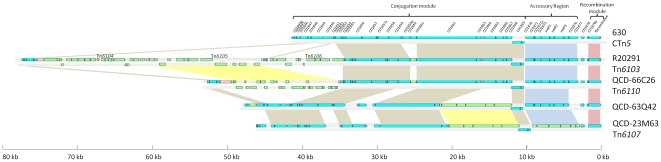
Schematic representation of CTn*5*-like elements in *C. difficile* strains. Blue ORFs are present in strain 630 CTn*5*. Green ORFs are not present in CTn*5*. Coloured boxes show regions of homology: red boxes show recombination modules, blue boxes show accessory genes, brown boxes show conjugation modules, yellow boxes show homologues that are not present in CTn*5* in strain 630. The element shown for strain QCD-66C26 is representative of elements identified in strains QCD-32G58 and QCD-37X79.

Several variants of CTn*5* were found in the *C. difficile* genomes that were examined. Strains QCD-37X79, QCD-66C26 and QCD-32G58 (all ribotype 027) contain an element 99% identical at the nucleotide level to the CTn*5*-like element Tn*6103* in R20291. However, in all three strains only Tn*6105* is present in the homologue of R20291 ORF 1743, and Tn*6104* and *Tn6106* are absent (Figure 8). We have demonstrated excision of the elements in strains QCD-66C26 and QCD-32G58, (Figure 6), and the elements were designated Tn*6110* and Tn*6111*, respectively. Although the element in R20291 has inserted in the same target site as CTn*5* in strain 630 (within a homologue of CD1844), in strains QCD-66C26 and QCD-32G58 it has inserted at a different site (between homologues of CD3369 and CD3393 encoding a hypothetical protein and putative RNA methyltransferase, respectively). Interestingly this is the same target site occupied by CTn*7* in 630. Tn*6110* and Tn*6111* are flanked by 5-bp sequences identical to those of CTn*5* in 630 and Tn*6103* in R20291 (Figure 6c, d). Although the genome of QCD-32G58 was assembled, there are still gaps in the sequence and the contigs on which Tn*6111* is present have not been joined. However, the fact that the circular form of the element and the empty target site were detected indicates that a functional element is present.

Strain QCD-63Q42 contains an element with a similar structure to CTn*5* in strain 630 (Figure 8), including the accessory module. However, the region homologous to CD1863 through to CD1870, encoding conjugation functions in CTn*5* in strain 630, has been replaced with a region containing several hypothetical genes and genes encoding restriction modification proteins. Excluding the insertion, the CTn*5*-like element in strain QCD-63Q42 shares on average 88% identity with CTn*5* in 630 and is inserted in the target site of CTn*7*, between homologs of CD3369 and CD3392, a hypothetical gene and a gene encoding a putative RNA methyltransferase, respectively. A CTn*7*-like element is present in tandem with the CTn*5*-like element in this strain. This element shares 97% nucleotide identity with CTn*7* of strain 630 although it has a 0.8-kb insertion containing a predicted transmembrane protein intergenic between the homologues of CD3389 and CD3390.

An element related to the CTn*5*-like element in strain QCD-63Q42, named here Tn*6107*, is present in strain QCD-23M63 (at least 80% sequence identity to CTn*5* excluding inserted region and accessory module) (Figure 8). In common with the CTn*5*-like element in QCD-63Q42, the conjugation region (CD1863–CD1870) has partially been replaced with a segment containing several genes encoding either hypothetical or restriction modification proteins. In addition, the accessory module is replaced with genes encoding hypothetical proteins, putative transcriptional regulators and ABC-transporters (see Table S1). The element is present between homologues of CD3369 and CD3393, the target site of CTn*7* in 630. The joint of the circular form and empty target site have 7-bp imperfect repeats, one copy of each is present on either side of the element in the integrated state (Figure 6b).

### CTn*4*-like elements

**Figure 9:**
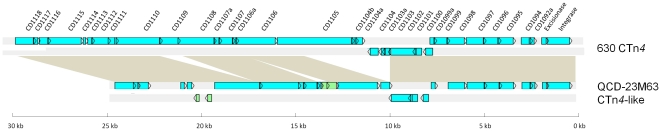
Schematic representation of the CTn*4*-like element in QCD-23M63. Blue ORFs are present in strain 630 CTn*4*, green ORFs are not present in CTn*4*. Brown boxes show regions of homology.

Strain QCD-23M63 (ribotype 078) contains a CTn*4*-like element (Figure 9) that has between 95 and 98% sequence identity with the element in strain 630 but with a deletion of ORFs CD1103–CD1105 (6 ORFs in total) and an insertion between the homologues of CD1106a and CD1107 consisting of genes encoding a putative histone acetyltransferase (ORF 4895) and a hypothetical protein. The element is inserted in the homologue of CD1036 in 630, a putative cell surface protein.

### Putative mobilisable transposons

**Figure 10:**
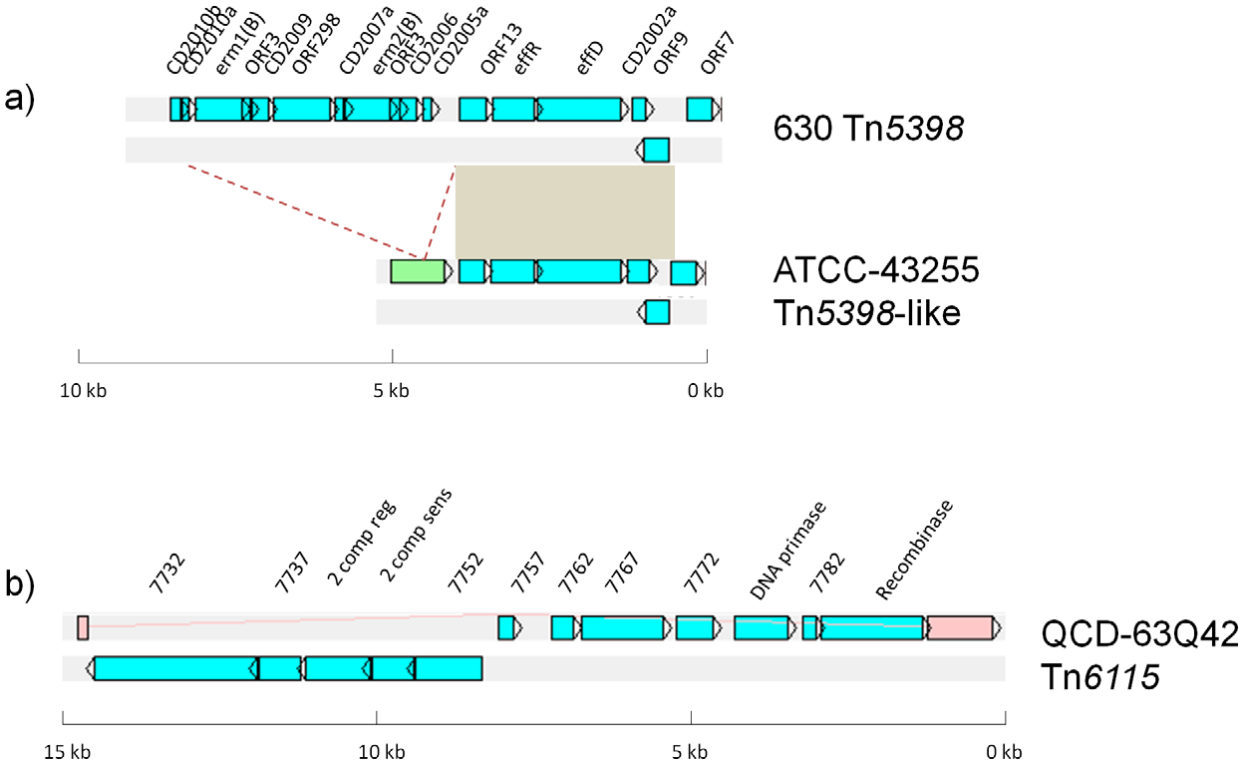
Schematic representation of the putative mobilisable elements. a) Comparison of Tn*5398* in strain 630 and the novel putative element in strain ATCC-43255. Blue ORFs are present in Tn*5398* in strain 630, green ORFs are not present in Tn*5398*. The insertion site of the *erm(B)* cassette is shown by dotted lines. Brown boxes show regions of homology. b) Representation of the novel putative mobilisable element in strain QCD-63Q42, Tn*6115*. Blue ORFs are part of the element, the pink ORF is the insertion site, a homologue of CD1573 in strain 630.

Strain ATCC 43255 contains a putative mobilisable transposon highly related to Tn*5398* in strain 630 [10] (99% nucleotide sequence identity excluding the *erm*B cassette (see below)) and present in an identical target site (Figure 10a). The sequence between the two direct repeats of the *erm*B cassette in Tn*5398* is not present in strain ATCC 43255, however, in its place is an ORF encoding a protein that is predicted to be secreted by virtue of an N-terminal export signal.

Strain QCD-63Q42 (ribotype 001) contains a novel 15-kb putative mobilisable transposon, designated Tn*6115* (Figure 10b), encoding several hypothetical gene products, a predicted ABC transporter (ORFs 7732 and 7737), a protein containing a predicted virulence-associated E domain [PFAM: PF05272] (ORF 7777) and a two component system (ORFs 7742 and 7747). A serine recombinase is predicted to be responsible for the potential excision of this element which is flanked by a GG dinucleotide direct repeat.

### Predicted functions of transposon encoded accessory proteins

In an effort to predict the role of the accessory regions of the conjugative transposons in the biology of *C. difficile*, we carried out a bioinformatics analysis of the predicted gene products encoded in the accessory regions using PSI-BLAST [22]. A limitation of PSI-BLAST is that the hits are listed according to their mathematical scores and not according to biological function. Therefore, we applied BYPASS, a program that uses fuzzy logic to rearrange the output from PSI-BLAST, putting in top position proteins with additional similarity in hydropathic profile, flexibility profile, amino acid composition, and length of the matched amino acid stretch, parameters which contribute to the accuracy of the functional prediction [23], [24]. We then searched the BYPASS output for hits with experimental evidence of function. To corroborate the function suggested by this analysis, we used P-SORT [25], PRO-DOM [26] and SMART [27] to predict the cellular location, the presence of signatures of protein families and domains, transmembrane regions and secretion signals. The analysis identified new potential functions for several genes annotated in the 630 genome sequence as hypothetical proteins (Table S1).

The analysis suggests that most of the accessory genes on the CTns in strain 630 encode ABC transporters and efflux systems which may function in resistance to antimicrobial peptides. For example, CD0363–0365 on CTn*1* encodes a predicted ABC transporter consisting of two different transmembrane domains and two ATP-binding domains each present on individual polypeptides. CD1095–1097 on CTn*4* encodes a predicted transporter consisting of two different transmembrane domains and a single ATP-binding domain. Experimental evidence of function is available for two hits in the BYPASS analysis, the plasmid-encoded BcrA and B proteins which comprise an ABC transporter mediating bacitracin resistance in *Enterococcus faecalis* [28]. The ATP-binding component, BcrA shares 52% identical amino acids with both CD0366 and CD1097, whereas the transmembrane protein BcrB shares 21% sequence identity with CD0365, CD1095 and CD1096. The ATP-binding domain of the ABC transporter (CD1349) mediating resistance to the cationic antimicrobial peptides, nisin and gallidermin [29] shares 32–36% amino acid identity with the predicted ATP-binding subunits encoded by CD0366 and CD1097 and the predicted transmembrane domains encoded by CD0365 and CD1095 share 15–22% identity with CD1350. The similarity with functionally characterised ABC transporters suggests that CD0363–0365 on CTn*1* and CD1095–1097 on CTn*4* may be involved in the export of antimicrobial peptides, a function likely to be important for intestinal colonisation.

Other accessory proteins carried by CTns in 630 that may function in interaction with the human host include a protein predicted to be surface-located by virtue of the presence of an N-terminal signal sequence and a C-terminal LPXTG membrane-anchoring domain. This protein (encoded by CD0386 on CTn*1*) was incorrectly annotated in the 630 genome sequence as a “collagen binding protein” because of the presence of B region domains which are repeated 7 times in the *Staphylococcus aureus* collagen-binding surface protein, Cna [30]. The B regions do not bind collagen, however; this is the function of the A domain. The B domains in Cna are thought to serve as a stalk that projects the A region from the cell surface facilitating its interaction with collagen [31]. There are two B repeats predicted in CD0386, but no A domain, and no other ligand-binding domain is identifiable using prediction tools. Similar LPXTG-linked proteins containing Cna B repeats are present on the CTn*1*-like elements in R20291 (gene 3453, 100% identical) and on all the CTn*1*-like elements identified, as well as in CTn*7* in strain 630 (CD3392, 95% identical). Interestingly, a membrane-anchored protein containing eight Cna B-type domain repeats and a predicted intimin/invasion domain, suggestive of a function in adhesion, is present on an integrated conjugative element in a strain of *Streptococcus pyogenes* [32]. As well as genes encoding putative ABC transporters, the novel CTn*1*-like elements carry other accessory genes with the potential to influence the ability of *C. difficile* to adapt to the human host. The CTn*1*-like element in QCD-23M63 carries a gene (3082), the predicted product of which gives highly significant PSI-BLAST scores with proteins belonging to the family of N-terminal (Ntn) hydrolases that includes bile salt hydrolases, β-lactam acylases and N-acyl homoserine lactone acylases (Table S1), however none of the hits identified by BYPASS are experimentally verified. A gene product that is 71% identical to the product of 23M63_3082 is carried by CTn*6* in strain 630 (CD3331). Alignment of the predicted proteins of 23M63_3082 and 630_CD3331 with an experimentally proven conjugated bile acid hydrolase from *C. perfringens* [Swiss-Prot:P54965] [33] and with a known penicillin G amidase from *Bacillus sphaeroides* [Swiss-Prot:P12256] [34] shows low level identity (14% and 12% identical amino acids, respectively).

An intriguing finding of our computational analysis is that many of the accessory genes on CTns in *C. difficile* are predicted to encode proteins with sequence similarity to predicted sigma factors and include a ‘helix-turn-helix’ motif involved in binding the conserved −35 region of promoters in DNA [35](Table S1). This is the only recognisable domain in TcdR, which has been proven experimentally to function as an alternative sigma factor in toxin gene expression [36]. Other potential sigma factors containing this domain are present on the CTn*1*-like elements in strains 43255, R20291, 23M63 and 63Q42 and on the CTn*5*-like elements in R20291 and 23M63 (Table S1). It will be interesting to determine if these gene products are able to recruit core RNA polymerase and bind to promoters within the element and/or to promoters within the recipient genome, and whether this influences the recipient cell transcriptome. In the CTn*5*-like element in R20291 (Tn*6103*), there are three tandem genes predicted to encode sigma factors (R20291_1754, 1755, and 1756) and a fourth predicted sigma factor encoded by R20291_1773 (Figure 5).

In addition to these putative sigma factors, the CTn*5*-like element in R20291 contains three genes predicted to encode transcriptional regulators including two (R20291_1747 and 1780) that contain a predicted helix-turn-helix motifs of type HTH_XRE found in a family of DNA binding proteins that include a bacterial plasmid copy control protein and various bacteriophage transcription control proteins [PFAM:PF01381] and one of which (1747) is related (30% identity) to the predicted transcriptional repressor within the regulatory region of Tn*916* (Orf 9) [37]. In addition to these transcriptional regulators, the CTn*5*-like element in strain R20291 contains a predicted two-component system (R20291_1748 and 1749) as well as an additional orphan response regulator (R20291_1775). Although genes encoding putative transcriptional regulators do occur in the accessory regions of other CTns in *C. difficile* (for example, predicted transcriptional regulators on CTn*6* (CD3334), and CTn*7* (CD3376)), the presence of so many putative transcriptional regulators on a single element in strain R20291 is intriguing.

## Discussion

In this study, we have shown that *C. difficile* genomes contain novel putative conjugative and mobilisable transposons related to the elements that were previously described for strains 630 and R20291. A library of conserved sequences of recombinase genes was compiled from Genbank and used to search for putative recombinases in recently sequenced *C. difficile* genomes. Aligning contigs from these genome sequences with the genome sequences of strain 630 and R20291 showed that 18 novel putative elements were present in the 9 different genomes. Most of these elements have a similar structure to CTn*1* or CTn*5* of strain 630.

A recent comparative genomic hybridisation study by Marsden *et al* [38] of 94 clinical strains of various ribotypes isolated predominately in the UK and the Netherlands reported that CTn*1* was absent or highly divergent in the majority of ribotype 027 and 001 strains and in all ribotype 078 and 015 strains. However, this conclusion was based on probes specific for the divergent accessory module (Marsden, personal communication). In contrast, we show that the core regions of CTn*1*-like elements i.e. the conjugation and integration/excision modules are present in all the strains in our collection including all five recently isolated ribotype 027 strains. This underlines the need for care when making conclusions about the presence or absence of particular integrative elements in genomes. Given the modular nature of these elements and the fact that the accessory regions are often divergent, it is important to be clear which modules have been specifically tested for.

In a comparative genome analysis, Stabler *et al.* [12] previously reported two unique conjugative transposons in strain R20291 that were absent in strain 630. One of these transposons, referred to as CTn*027* by Stabler *et al.*, and renamed Tn*6103* here, was reported to contain a single 20-kb phage island which they termed SMPI. We have shown that, rather than a single large insertion, Tn*6103* contains three distinct insertions which are likely to be mobilisable transposons and therefore they have been named Tn*6104*, Tn*6105* and Tn*6106*. We have demonstrated excision of Tn*6104* and shown that some ribotype 027 strains contain an element that is related to Tn*6103* but lacks the Tn*6104* and Tn*6106* insertions.

Excision from the genome to a circular intermediate is a prerequisite for conjugal transfer [39]. Circular molecules were demonstrated in this study for several of the previously described elements as well as for some of the novel elements. To determine if the elements were capable of conjugal transfer, they were marked with an antibiotic resistance gene and conjugative transfer of CTn*1*, CTn*2*, CTn*4*, CTn*5* and CTn*7* from strain 630Δ*Erm* to CD37 was demonstrated. Although we detected a circular form of Tn*6103* in R20291, transfer of this element to CD37 was not demonstrated. This is possibly because of the insertion of the mobilizable transposons, Tn*6104*, Tn*6105* and Tn*6106* in the conjugation module.

It is interesting to note that many of the putative mobile elements have been conserved, with many exhibiting variation only in the module of accessory genes. We have attempted to gain insight into the functions of the genes in these modules using a computational approach. Our analysis suggests that the majority of accessory genes carried on CTns in *C. difficile* encode ABC transporters and efflux systems presumed to function in resistance to antimicrobial peptides, produced either by the host innate immune response, or by microbial competitors in the intestinal niche. In addition, we have shown that some elements carry genes with the potential to encode bile salt hydrolases which could contribute to the ability of the bacterium to adapt to the human host. A secreted protein which appears to have a stalk-like structure that projects it away from the cell surface is also worthy of further investigation since it is likely to be involved in the interaction with the human host. Perhaps the most interesting finding of our study is that several of the CTns encode sigma factor-like proteins and transcriptional regulators.

Investigating when both the accessory proteins and also the excision and transfer proteins are expressed will be the next step in understanding the function and regulation of these elements. We are currently using RT-PCT to investigate the conditions under which the putative surface protein, CD0386, is expressed.

Acquisition of mobile genetic elements will result in numerous heritable changes, over and above the addition of new genes. Insertion between ORFs may result in transcriptional effects in the locality of the insertion site which can fundamentally alter the phenotype of the host. Elements can insert into ORFs resulting in gene inactivation, and we have shown here that similar elements select different target sites in different strains, e.g. the CTn*1*-like elements. Gene fusion events may also occur, eg., CTn*5* promotes a fusion with CD1844 in strain 630 [8]. Furthermore the newly acquired DNA can be a substrate for recombination promoting more general genome rearrangements. Thus, it appears there is much still to learn about the contribution of mobile genetic elements to the biology of *C. difficile*.

## Materials and Methods

### Bacterial strains and culture conditions

The bacterial strains used in this study are listed in Table 2 and Table S2. *C. difficile* strains were grown on brain heart infusion (BHI) agar plates (Oxoid Ltd, Basingstoke, UK) supplemented with 5% defibrinated horse blood (E & O laboratories, Bonnybridge, UK) or in BHI broth (Oxoid Ltd). Cultures were grown at 37°C in anaerobic conditions (80% N_2_, 10% H_2_, 10% CO_2_). *E. coli* CA-434 was grown on Luria-Bertani agar plates (Sigma-Aldrich Company Ltd., Dorset, UK) at 37°C in aerobic conditions.

**Table 2:** Properties of *C. difficile* strains used in this study.

Strain	Ribotype*	Other strain information†	Place of isolation, date	Clinical details	Source
630	012	Tc^R^ Erm^R^	Zurich, Switzerland, 1982	pseudomembraneous colitis	[44]
R20291	027	Tc^S^ Erm^S^	Stoke Mandeville Hospital, UK, 2006		[45]
CD196	027		Paris, France, 1985		
ATCC43255 (VPI10463)	087				
QCD-23M63	078	Toxinotype V, Tc^S^ Erm^S^	Montreal, Quebec, Canada	Severe CDAD	Dr. A. Dascal
QCD-32G58	027	NAP1, Binary toxin +ve, *tcdC* 18 bp deletion, Tc^S^ Erm^R^	Quebec, Canada, 2004–8	CDAD	Dr. A. Dascal
QCD-37X79	027	NAP1a/001	London, Ontario Canada, 2005	Severe CDAD	Dr. A. Dascal
QCD-63Q42	001	NAP2	Quebec City, Quebec, Canada, 2005	Severe CDAD	Dr. A. Dascal
QCD-66C26	027	NAP1, Binary toxin +ve, tcdC delta-117 and 18 bp deletion, Tc^S^ Erm^R^	Quebec, Canada, 2004–8	CDAD	Dr. A. Dascal
QCD-76W55	027	NAP1	Minnesota, Minneapolis, US, 1988		Dr. A. Dascal
QCD-97B34	027	NAP1b/006	St. John's, Newfoundland, Canada, 2004	Severe CDAD	Dr. A. Dascal

Tc: tetracycline, Erm: erythromycin, ^S^: sensitive, ^R^: resistant.

*not determined, ribotype equivalent,

† Toxinotype [46]; NAP. North American PFGE type [4]; *tcdC* 18 bp deletion, tcdC delta-117 [47].

### DNA preparation and PCR analysis

DNA was isolated using the Puregene yeast/bacterial kit B (Qiagen, Crawley, UK) according to the manufacturer's instructions, with the addition of 3 µl of both the lytic enzyme solution and RNAse A solution instead of 1.5 µl at the appropriate steps in the protocol. Purity assessment and quantification was done using a Nanodrop 1000 spectrophotometer.

PCR amplifications were carried out using the NEB *Taq* Polymerase kit (New England Biolabs, Herts, UK) according to the manufacturer's instructions with 10 mM dNTPs (NEB). The primers that were used are listed in Table S3 (Sigma-Genosys, UK).

PCR products were run on a 1% agarose gel at 100 mV for 1 hour, supplemented with Gelred at a 1∶10,000 dilution (Biotium, Hayward, USA). PCR products were purified with the spin column PCR purification kit (NBS Biologicals ltd, Cambridgeshire, UK) according to the manufacturer's instructions. When multiple PCR products were present, the product was purified using the spin column gel extraction kit according to the manufacturer's instructions.

PCR products were sequenced at the Department of Biochemistry, University of Cambridge.

### PCR analysis of transposon excision and identification of transposon ends

In order to investigate if a putative element can excise form the genome, PCR analysis was performed to amplify the joint region of the circular intermediate using primers at the ends of the element, facing outward. The sequence of joint regions of novel circular intermediates was deposited in Genbank; accession numbers are provided in Table 1. Additionally, the regenerated target sites and the junctions between the element and the genome were amplified. Comparison of the sequences of the junctions between the genome and element, the empty target site in the genome and the circular joint of the excised molecule enabled identification of the ends of the transposons.

### ClosTron retargeting

#### Clostron Targets CD0364, CD0386, CD3392, CD1873

The ClosTron system was used to make insertions in strain 630 ORFs CD0364 and CD0386 (CTn*1*) and CD3392 (CTn*7*) by retargeting a group II intron as described by Heap *et al.* [19]. Suitable target sites were identified and primers were designed using the Targetron Gene Knockout System kit (Sigma-Aldrich) (primers listed in Table S4). Splicing by Overlapping Extension PCR was used to create the specific intron retargeting sequence which was cloned into pMTL-007. The plasmids were transferred from *E. coli* CA-434 into *C. difficile* 630Δ*erm* by conjugation. Selection was carried out on agar containing thiamphenicol (Sigma-Aldrich) (15 µg/ml) and *C. difficile* selective supplement (Oxoid Ltd). Thiamphenicol resistant colonies were suspended in BHI broth containing 1 mM IPTG (Sigma-Aldrich) and incubated for 3 hours at 37°C. Cultures were spread onto BHI plates containing 40 µg/ml lincomycin (Sigma-Aldrich) and *C. difficile* selective supplement. Colonies were restreaked onto fresh selective plates and the insertions were confirmed using PCR to amplify the junction between the target site and the intron.

#### Clostron targets CD0428, CD1099 and R20291_1803

The revised ClosTron system [40] was used to target 630 ORFs CD0428 (CTn*2*) and CD1099 (CTn*4*) and R20291 ORF 1803 (Tn*6103*). The construction of the plasmid for the revised protocol varies in that the target sites were identified using the algorithm available at www.clostron.com. The plasmid was produced by DNA2.0 (Menlo Park, USA). Colonies on plates containing thiamphenicol and *C. difficile* selective supplement were streaked directly onto plates containing lincomycin and *C. difficile* selective supplement. Insertions were confirmed using PCR as described above.

### Filter matings

Filter matings were carried out as described previously [41]. Putative transconjugants were screened with ErmRAM primers [19] to confirm the presence of the marked elements. To confirm the identity of the recipient strain, PCR with primers Lok1 and Lok3 was used to confirm the absence of the PaLoc [42]. Transfer frequencies were calculated as number of transconjugants per donor cell.

### Construction of the *in silico* recombinase library

The nucleotide sequences of the genes encoding serine or tyrosine-based recombinases associated with conjugative transposons, plus those phylogenetically related genes present on bacterial genomes, were downloaded from Genbank at the NCBI. This included sequences from the following (the numbers in box brackets are the genome position while those in parentheses are the Genbank accession numbers): Tn*916* (U09422), Tn*1545* (X61025), Tn*1549* (AF192329), Tn*5382* (AF063010), Tn*5386* (DQ321786), *Streptococcus thermophilus* genomic island CIME19258 [553–1749bp] (AJ586571), *C. difficile* 630 [1284507–1285700 bp] (AM180355), Tn*4451* (U15027), *Treponema denticola* ATCC 35405 [2204491–2206329 bp] (NC_002967), *Campylobacter coli* RM2228 [3327–5213] (NZ_AAFL01000021), *Enterococcus faecalis* V583 [2204960–2206573 bp] (NC_004668). *Streptococcus pyogenes* MGAS2096 [1092958–1094889 bp] (CP000261), *Streptococcus suis* [58479–58655 bp] (NZ_AAFA02000004).

### Transposon nomenclature

Novel transposons were named according to the transposon registry guidelines [15]. The registry stipulates that if functionality of a transposable element is demonstrated e.g. by excision from the host genome, or the entire sequence of a putative transposable element is determined and shown to be <100% identical to previously known transposable elements, then a Tn number is warranted [15].

### Sequence alignments and comparisons

All *C. difficile* genome sequences available in the database at NCBI as of March 2010 were searched using the BLAST algorithm with the 33 sequences of the recombinase library as input. Data in this paper was updated for all genome corrections made, up until October 2010. Additionally, the sequences of the ORFs of the (putative) conjugative transposons of strain 630, CTn*1*, CTn*2*, CTn*4*, CTn*5* and CTn*7* were used in this search. All contigs containing a putative recombinase were compared to the genome sequence of strains 630 and R20291 to look for insertions. Comparisons were made using Doubleact [43] and visualised using the Artemis Comparison Tool [20].

### Sequence annotation

Predicted proteins present on putative conjugative transposons that had previously been annotated as hypothetical genes were analysed using several bioinformatics tools [24]: PSI-BLAST [22], BYPASS [23], P-SORT [25], PRODOM ([26] and SMART [27].

PSI-BLAST was performed and the PSSM matrix after the fifth iteration, or when the program converged from lack of further similarities, was used for analysis with BYPASS. In parallel, analysis of the protein sequences was performed with the P-SORT, PRO-DOM and SMART programs.

## Supporting Information

Table S1 **Results of BYPASS, PSORT, SMART and PRODOM searches of hypothetical proteins.**
(XLS)

Table S2 **Bacterial strains and plasmids produced in this study.**
(PDF)

Table S3 **PCR primers used to amplify junctions of circular intermediates of conjugative transposons and empty target sites.** PCR primers used to produce ClosTron mutants, and to screen transconjugant cells.
(PDF)

Table S4 **PCR primers used to produce ClosTron mutants, and to screen transconjugant cells.**
(PDF)

## References

[1] Bartlett JG (2008). Historical perspectives on studies of *Clostridium difficile* and *C. difficile* infection.. Clin Infect Dis.

[2] Borriello SP (1998). Pathogenesis of *Clostridium difficile* infection.. J Antimicrob Chemother.

[3] Kuehne SA, Cartman ST, Heap JT, Kelly ML, Cockayne A (2010). The role of toxin A and toxin B in *Clostridium difficile* infection.. Nature.

[4] Gal M, Northey G, Brazier JS (2005). A modified pulsed-field gel electrophoresis (PFGE) protocol for subtyping previously non-PFGE typeable isolates of *Clostridium difficile* polymerase chain reaction ribotype 001.. J Hosp Infect.

[5] Freeman J, Bauer MP, Baines SD, Corver J, Fawley WN (2010). The changing epidemiology of *Clostridium difficile* infections.. Clin Microbiol Rev.

[6] Bauer MP, Veenendaal D, Verhoef L, Bloembergen P, van Dissel JT (2009). Clinical and microbiological characteristics of community-onset *Clostridium difficile* infection in The Netherlands.. Clinical Microbiology and Infection.

[7] Brazier JS, Raybould R, Patel B, Duckworth G, Pearson A (2008). Distribution and antimicrobial susceptibility patterns of *Clostridium difficile* PCR ribotypes in English hospitals, 2007–08.. Euro Surveill.

[8] Sebaihia M, Wren BW, Mullany P, Fairweather NF, Minton N (2006). The multidrug-resistant human pathogen *Clostridium difficile* has a highly mobile, mosaic genome.. Nat Genet.

[9] Mullany P, Wilks M, Lamb I, Clayton C, Wren B (1990). Genetic analysis of a tetracycline resistance element from *Clostridium difficile* and its conjugal transfer to and from *Bacillus subtilis*.. J Gen Microbiol.

[10] Mullany P, Wilks M, Tabaqchali S (1995). Transfer of macrolide-lincosamide-streptogramin B (MLS) resistance in *Clostridium difficile* is linked to a gene homologous with toxin A and is mediated by a conjugative transposon, Tn*5398*.. J Antimicrob Chemother.

[11] He M, Sebaihia M, Lawley TD, Stabler RA, Dawson LF (2010). Evolutionary dynamics of *Clostridium difficile* over short and long time scales.. Proc Natl Acad Sci U S A.

[12] Stabler RA, He M, Dawson L, Martin M, Valiente E (2009). Comparative genome and phenotypic analysis of *Clostridium difficile* 027 strains provides insight into the evolution of a hypervirulent bacterium.. Genome Biol.

[13] Clewell DB, Flannagan SE, Jaworski DD (1995). Unconstrained Bacterial Promiscuity - the Tn*916*-Tn*1545* Family of Conjugative Transposons.. Trends in Microbiology.

[14] Roberts AP, Mullany P (2009). A modular master on the move: the Tn*916* family of mobile genetic elements.. Trends Microbiol.

[15] Roberts AP, Chandler M, Courvalin P, Guedon G, Mullany P (2008). Revised nomenclature for transposable genetic elements.. Plasmid.

[16] Abraham LJ, Rood JI (1987). Identification of Tn*4451* and Tn*4452*, chloramphenicol resistance transposons from *Clostridium perfringens*.. J Bacteriol.

[17] Lyras D, Adams V, Lucet I, Rood JI (2004). The large resolvase TnpX is the only transposon-encoded protein required for transposition of the Tn*4451*/*3* family of integrative mobilizable elements.. Mol Microbiol.

[18] Bannam TL, Crellin PK, Rood JI (1995). Molecular genetics of the chloramphenicol-resistance transposon Tn*4451* from *Clostridium perfringens*: the TnpX site-specific recombinase excises a circular transposon molecule.. Mol Microbiol.

[19] Heap JT, Pennington OJ, Cartman ST, Carter GP, Minton NP (2007). The ClosTron: a universal gene knock-out system for the genus *Clostridium*.. J Microbiol Methods.

[20] Carver T, Berriman M, Tivey A, Patel C, Bohme U (2008). Artemis and ACT: viewing, annotating and comparing sequences stored in a relational database.. Bioinformatics.

[21] Haraldsen JD, Sonenshein AL (2003). Efficient sporulation in *Clostridium difficile* requires disruption of the *sigmaK* gene.. Mol Microbiol.

[22] Altschul SF, Madden TL, Schaffer AA, Zhang J, Zhang Z (1997). Gapped BLAST and PSI-BLAST: a new generation of protein database search programs.. Nucleic Acids Res.

[23] Gomez A, Cedano J, Espadaler J, Hermoso A, Pinol J (2008). Prediction of protein function improving sequence remote alignment search by a fuzzy logic algorithm.. Protein J.

[24] Hernandez S, Gomez A, Cedano J, Querol E (2009). Bioinformatics annotation of the hypothetical proteins found by omics techniques can help to disclose additional virulence factors.. Curr Microbiol.

[25] Nakai K, Kanehisa M (1992). A knowledge base for predicting protein localization sites in eukaryotic cells.. Genomics.

[26] Wootton JC, Federhen S (1996). Analysis of compositionally biased regions in sequence databases.. Methods Enzymol.

[27] Schultz J, Milpetz F, Bork P, Ponting CP (1998). SMART, a simple modular architecture research tool: identification of signaling domains.. Proc Natl Acad Sci U S A.

[28] Manson JM, Keis S, Smith JM, Cook GM (2004). Acquired bacitracin resistance in *Enterococcus faecalis* is mediated by an ABC transporter and a novel regulatory protein, BcrR.. Antimicrob Agents Chemother.

[29] McBride SM, Sonenshein AL (2011). Identification of a genetic locus responsible for antimicrobial peptide resistance in *Clostridium difficile*.. Infect Immun.

[30] Patti JM, Jonsson H, Guss B, Switalski LM, Wiberg K (1992). Molecular characterization and expression of a gene encoding a *Staphylococcus aureus* collagen adhesin.. J Biol Chem.

[31] Rich RL, Demeler B, Ashby K, Deivanayagam CC, Petrich JW (1998). Domain structure of the *Staphylococcus aureus* collagen adhesin.. Biochemistry.

[32] Beres SB, Musser JM (2007). Contribution of exogenous genetic elements to the group A *Streptococcus* metagenome.. PLoS One.

[33] Coleman JP, Hudson LL (1995). Cloning and characterization of a conjugated bile acid hydrolase gene from *Clostridium perfringens*.. Appl Environ Microbiol.

[34] Olsson A, Uhlen M (1986). Sequencing and heterologous expression of the gene encoding penicillin V amidase from *Bacillus sphaericus*.. Gene.

[35] Zafar MA, Sanchez-Alberola N, Wolf RE (2011). Genetic Evidence for a Novel Interaction between Transcriptional Activator SoxS and Region 4 of the sigma(70) Subunit of RNA Polymerase at Class II SoxS-Dependent Promoters in *Escherichia coli*.. J Mol Biol.

[36] Mani N, Dupuy B (2001). Regulation of toxin synthesis in *Clostridium difficile* by an alternative RNA polymerase sigma factor.. Proc Natl Acad Sci U S A.

[37] Flannagan SE, Zitzow LA, Su YA, Clewell DB (1994). Nucleotide sequence of the 18-kb conjugative transposon Tn*916* from *Enterococcus faecalis*.. Plasmid.

[38] Marsden GL, Davis IJ, Wright VJ, Sebaihia M, Kuijper EJ (2010). Array comparative hybridisation reveals a high degree of similarity between UK and European clinical isolates of hypervirulent *Clostridium difficile*.. BMC Genomics.

[39] Scott JR, Kirchman PA, Caparon MG (1988). An intermediate in transposition of the conjugative transposon Tn*916*.. Proc Natl Acad Sci U S A.

[40] Heap JT, Kuehne SA, Ehsaan M, Cartman ST, Cooksley CM (2009). The ClosTron: Mutagenesis in *Clostridium* refined and streamlined.. J Microbiol Methods.

[41] Hussain HA, Roberts AP, Mullany P (2005). Generation of an erythromycin-sensitive derivative of *Clostridium difficile* strain 630 (630Deltaerm) and demonstration that the conjugative transposon Tn*916*DeltaE enters the genome of this strain at multiple sites.. J Med Microbiol.

[42] Braun V, Hundsberger T, Leukel P, Sauerborn M, von Eichel-Streiber C (1996). Definition of the single integration site of the pathogenicity locus in *Clostridium difficile*.. Gene.

[43] Underwood A, Green J http://www.hpa-bioinfotools.org.uk/pise/double_act.html.

[44] Wust J, Hardegger U (1983). Transferable resistance to clindamycin, erythromycin, and tetracycline in *Clostridium difficile*.. Antimicrob Agents Chemother.

[45] Stabler RA, Gerding DN, Songer JG, Drudy D, Brazier JS (2006). Comparative phylogenomics of *Clostridium difficile* reveals clade specificity and microevolution of hypervirulent strains.. J Bacteriol.

[46] Rupnik M, Avesani V, Janc M, von Eichel-Streiber C, Delmee M (1998). A novel toxinotyping scheme and correlation of toxinotypes with serogroups of *Clostridium difficile* isolates.. J Clin Microbiol.

[47] MacCannell DR, Louie TJ, Gregson DB, Laverdiere M, Labbe AC (2006). Molecular analysis of *Clostridium difficile* PCR ribotype 027 isolates from Eastern and Western Canada.. J Clin Microbiol.

