# Supplementary material for: Genetic Organisation, Mobility and Predicted Functions of Genes on Integrated, Mobile Genetic Elements in Sequenced Strains of Clostridium difficile
Source: PLoS One. 2011 Aug 18;6(8):e23014. doi: 10.1371/journal.pone.0023014 (PMC3158075; doi:10.1371/journal.pone.0023014)
Supplement: Table S2 — Bacterial strains and plasmids produced in this study. (PDF) [file pone.0023014.s002.pdf]

**Table S2; Bacterial strains and plasmids produced in this study**

| **Strain or plasmid** | **Properties** | **Reference** |
|---|---|---|
| *Strain* | | |
| *C. difficile* 630ΔErm | $Tc^R$ $Linc^S$ $Rif^S$ | [1] |
| *C. difficile* CD37 | $Tc^S$ $Linc^S$ $Rif^R$, Ribotype 009 | [2] |
| *E. coli* CA434 | | |
| *C. difficile* 630ΔErm-CD0364::ErmRAM | ClosTron insertion in CD0364 | This study |
| *C. difficile* 630ΔErm-CD0386::ErmRAM | ClosTron insertion in CD0386 | This study |
| *C. difficile* 630ΔErm-CD0428::ErmRAM | ClosTron insertion in CD0428 | This study |
| *C. difficile* 630ΔErm-CD1099::ErmRAM | ClosTron insertion in CD1099 | This study |
| *C. difficile* 630ΔErm-CD1873::ErmRAM | ClosTron insertion in CD1873 | This study |
| *C. difficile* 630ΔErm-CD3392::ErmRAM | ClosTron insertion in CD3392 | This study |
| *Plasmid* | | |
| pMTL007 | $Cm^R$, $Linc^R$ | Heap 2007 |
| pMTL007-CD0364 | pMTL007, RAM retargeted to CD0364 | This study |
| pMTL007-CD0386/CD3392 | pMTL007, RAM retargeted to CD0386/CD3392 | This study |
| pMTL007C-E5-CD0428 | pMTL007, RAM retargeted to CD0428 | This study |
| pMTL007C-E5 CD1099 | pMTL007, RAM retargeted to CD1099 | This study |
| pMTL007-CD1873 | pMTL007, RAM retargeted to CD1873 | This study |

Tc: tetracycline, Linc: lincomyin, Rif: Rifampicin, Cm: chloramphenicol, $^R$: resistant, $^S$: sensitive

**References**


1. Hussain HA, Roberts AP, Mullany P (2005) Generation of an erythromycin-sensitive derivative of *Clostridium difficile* strain 630 (630Deltaerm) and demonstration that the conjugative transposon Tn*916*DeltaE enters the genome of this strain at multiple sites. J Med Microbiol 54: 137-141.
2. Hachler H, Kayser FH, Berger-Bachi B (1987) Homology of a transferable tetracycline resistance determinant of *Clostridium difficile* with *Streptococcus* (*Enterococcus*) *faecalis* transposon Tn*916*. Antimicrob Agents Chemother 31: 1033-1038.
