# Supplementary material for: Genetic Organisation, Mobility and Predicted Functions of Genes on Integrated, Mobile Genetic Elements in Sequenced Strains of Clostridium difficile
Source: PLoS One. 2011 Aug 18;6(8):e23014. doi: 10.1371/journal.pone.0023014 (PMC3158075; doi:10.1371/journal.pone.0023014)
Supplement: Table S3 — PCR primers used to amplify junctions of circular intermediates of conjugative transposons and empty target sites. PCR primers used to produce ClosTron mutants, and to screen transconjugant cells. (PDF) [file pone.0023014.s003.pdf]

Table S3; PCR primers used to amplify junctions of circular intermediates of conjugative transposons and empty target sites

Primer	Sequence 5'- 3'
CTn1 LEO	GGGCTTATCAAGCAATCCAA
CTn1 REO	GAAATCTGGTCGGCACTCAT
CTn1 TSF	GCAAAAGTTGCAGGAATCAT
CTn1 TSR	GAGAAAATGGCTCTGCTATGC
CTn2 LEO	CGAAATTGATCCTGCAATGA
CTn2 REO	GTTCCAAGTCCGTTTTCGCT
CTn2 TSF	ACCCTCCTAGACCAGGCATT
CTn2 TSR	CAACATGAGAAACCAGACGGTA
CTn3 LEO	CCACTTGATATGAAAAATCAAATGGCTC
CTn3 REO	CCGTTCTATGCTGTGTGAGCTG
CTn3 TSF	GGCGAAAACTGCTTGGATT
CTn3 TSR	CCTTTGTTTTCTACATCAAGTATAGCC
CTn4 LEO	TGAACCCCAAAGCATTGCAG
CTn4 REO	TGGTGGTTGCTCATAACCG
CTn4 TSF	GGATAATGCAATAGAAGCATGTG
CTn4 TSR	CATATCACCTTCCTATCCAATTCTT
CTn5 LEO	CATCCCACCTGATAGGCAAG
CTn5 REO	GATGCCGTTGCTGAGGTAAT
CTn5 TSF	GCGCCATTGGAAATGACTAT
CTn5 TSR	CCAGTCCATGGTTGAAAAGTT
CTn6 LEO	CCAAAGACGCAAGCCAGTAT
CTn6 REO	CGGCAGGGAGGATAACTACA
CTn6 TSF	CGCTCTTGCCCATATTTCAG
CTn6 TSR	CGCATCAGGAACATTGACTA
CTn7 LEO	GACCCTGCACAGTTGACCTT
CTn7 REO	CTTGTGAGCTTCGCCATACT
CTn7 TSF	CCGAAATCGCTTTACAGTGG
CTn7 TSR	CCCACCTAGAAAGGGATGTG
R20291 CTn1 LEO	GTGAGGGATTGCCAAAGGTA
R20291 CTn1 REO	CGGCACTCATACGGGTACTT
R20291 CTn1 TSF	GATTCGTTAAATGCCTCTTTATTG
R20291 CTn1 TSR	GAGTGAAGAAGACTGGTTGACAGA
R20291 CTn5 LEO	CCAACCATTGGTATTCCGTA
R20291 CTn5 REO	GATGCCGTTGCTGAGGTAAT
R20291 CTn5 TSF	GCGCCATTGGAAATGACTAT
R20291 CTn5 TSR	CCAGTCCATGGTTGAAAAGTT
23M63 CTn1 LEO	GAAGCCCGTACACAATCGTT
23M63 CTn1 REO	GGGCTTATCAAGCAATCCAA
23M63 CTn1 TSF	GGCTTCTTTAATTTGCTCTTTA
23M63 CTn*1* TSR	GGTTGTGTTTGCAGCTGTTA
23M63 CTn5 LEO	CGCCTTTGGTAACCATTTGT
23M63 CTn5 REO	GCATTGAAGAAAAGGCTTGAG

LEO = Left End Out; REO = Right End Out; TSF = Target Site Forward; TSR = Target Site Reverse.
