# Supplementary material for: Genetic Organisation, Mobility and Predicted Functions of Genes on Integrated, Mobile Genetic Elements in Sequenced Strains of Clostridium difficile
Source: PLoS One. 2011 Aug 18;6(8):e23014. doi: 10.1371/journal.pone.0023014 (PMC3158075; doi:10.1371/journal.pone.0023014)
Supplement: Table S4 — PCR primers used to produce ClosTron mutants, and to screen transconjugant cells. (PDF) [file pone.0023014.s004.pdf]

**Supplementary table 4; PCR primers used to produce ClosTron mutants, and to screen transconjugant cells**

| Primer | Sequence 5'-3' |
|---|---|
| *Intron retargeting* | |
| EBS universal | CGAAATTAGAAACTTGCGTTCAGTAAAC |
| IBS CD0364 | AAAAAAGCTTATAATTATCCTTATGACACAGAAATGTGCGCCCAGATAGGGTG |
| EBS1d CD0364 | CAGATTGTACAAATGTGGTGATAACAGATAAGTCAGAAATGTTAACTTACCTTTCTTTGT |
| IBS CD0386/CD3392 | AAAAAAGCTTATAATTATCCTTAGTCATCCATTACGTGCGCCCAGATAGGGTG |
| EBS1d CD0386/CD3392 | CAGATTGTACAAATGTGGTGATAACAGATAAGTCCATTACTTTAACTTACCTTTCTTTGT |
| EBS2 CD0386/CD3392 | TGAACGCAAGTTTCTAATTTCGATTATGACTCGATAGAGGAAAGTGTCT |
| IBS CD1873 | AAAAAAGCTTATAATTATCCTTATAAAACATTTACGTGCGCCCAGATAGGGTG |
| EBS1d CD1873 | CAGATTGTACAAATGTGGTGATAACAGATAAGTCATTTACCATAACTTACCTTTCTTTGT |
| EBS2 CD1873 | TGAACGCAAGTTTCTAATTTCGGTTTTTTATCGATAGAGGAAAGTGTCT |
| *Plasmid sequencing* | |
| pMTL007-R1 | AGGGTATCCCCAGTTAGTGTTAAGTCTTGG |
| 5402F-F1 | TTAAGGAGGTGTATTTCATATGACCATGATTACG |
| *Mutant screening* | |
| CD0364 TSF | GATTAAAGCCCTCGCACAAG |
| CD0364 TSR | CGTAACCAGCGGACACATTA |
| CD0386/CD3392 TSF | CCCAAAATGAAAGGAGCATT |
| CD0386/CD3392 TSR | GCGTCCGCTAATTTTGTGAT |
| CD1873 TSF | CAATAATGAAATCAAGCAATGAAA |
| CD1873 TSR | GCTTCGAGTGAAACAACATTCT |
| CD0428 TSF | CATGGAATCTTCGCATCAGA |
| CD0428 TSR | GCGAATGTCCTCACTAACCG |
| CD1099 TSF | GTCCATACCGGGCATCATAA |
| CD1099 TSR | CAGAAAGGACGGGTGTTGTT |
| ErmRAM-F | ACGCGTTATATTGATAAAAATAATAATAGTGGG |
| ErmRAM-R | ACGCGTGCGACTCATAGAATTATTTCCTCCCG |

| | |
|---|---|
| *Transconjugant screening* | |
| lok1 | AAAATATACTGCACATCTGTATAC |
| lok3 | TTTACCAGAAAAAGTAGCTTTAA |
